# FORG3D: Force-directed 3D graph editor for visualization of integrated genome scale data

**DOI:** 10.1186/1752-0509-3-26

**Published:** 2009-02-24

**Authors:** Jussi Paananen, Garry Wong

**Affiliations:** 1 A.I. Virtanen Institute of Molecular Sciences, University of Kuopio, Kuopio, Finland; 2 Department of Biosciences, University of Kuopio, P.O. Box 1627, 70211 Kuopio, Finland

## Abstract

**Background:**

Genomics research produces vast amounts of experimental data that needs to be integrated in order to understand, model, and interpret the underlying biological phenomena. Interpreting these large and complex data sets is challenging and different visualization methods are needed to help produce knowledge from the data.

**Results:**

To help researchers to visualize and interpret integrated genomics data, we present a novel visualization method and bioinformatics software tool called FORG3D that is based on real-time three-dimensional force-directed graphs. FORG3D can be used to visualize integrated networks of genome scale data such as interactions between genes or gene products, signaling transduction, metabolic pathways, functional interactions and evolutionary relationships. Furthermore, we demonstrate its utility by exploring gene network relationships using integrated data sets from a *Caenorhabditis elegans *Parkinson's disease model.

**Conclusion:**

We have created an open source software tool called FORG3D that can be used for visualizing and exploring integrated genome scale data.

## Background

To understand the biological phenomena behind systems biology data, researchers often need to combine different kinds of experimental results, creating complex data sets of integrated information. Systems biology efforts are directed towards acquisition of high-throughput-omics data and then analysis and modeling on a whole organism scale. Modeling provides the ability to infer functions and make predictions based on network perturbations [1-6]. Among the most commonly modeled biological data are protein-protein interactions. Proteins do not act alone, but in concert with other proteins and mapping their interactions can provide insight into the molecular pathways in which they participate [7]. Protein-protein interaction maps also indicate a high level of molecular connectivity between different biological pathways thus highlighting the inter-related functions of many biological processes [8]. When approaches to perturb network interactions are utilized, such as genetic interactions using RNAinterference, null-mutant alleles, or the two in combination, even greater knowledge on the identity of key network sites can be obtained [9-11]. Integration of protein-protein interaction data with transcriptomics data has also been successfully applied to differentiate permanent and transient cellular complexes [12]. Thus, the ability to construct, analyze, and interpret integrated-omics data is fundamental to understanding gene function in systems biology. To help researchers to visualize and interpret genome scale biology data, we present a novel visualization method that is based on real-time three-dimensional force-directed graphs that can be used in discovery of novel knowledge from the data.

Graphs are a natural choice for visualizing genome scale data, as many connections in biology can be thought as networks, for example interactions between genes or gene products, signal transduction, metabolic pathways, functional interactions and evolutionary relationships. In addition, virtually any kind of experimental data that describes correlations or distances between measurements can be presented as a graph. Commonly graphs consist of nodes and edges, where nodes present key elements (such as genes or proteins) and edges present connections between the elements. Visual appearance such as size, color and shape of nodes and edges can be changed to describe different features. With force-directed graphs, nodes and edges are not only assigned with visual attributes, but also with physical ones, such as mass and electric charge for nodes, and spring-constant for edges. The graph is then simulated as a physical model, where nodes interact with each other based on their physical attributes while edges constrain their movement. This allows for intuitive visualization of connection strength by the distance between the connected nodes. Force-directed graphs also provide a simple and effective solution to the complex challenge of arranging nodes and edges in a formation that is easily interpreted by a human. Different force-directed layouts have been successfully applied to visualizing biological data in the past [13,14].

To demonstrate the concept of real-time force directed three-dimensional graphs in visualization of integrated systems biology data and to provide researchers with practical software tool, we have developed a software program called FORG3D.

## Implementation

FORG3D is an open source software program for visualization of network data using three-dimensional force-directed graphs. FORG3D can be used as a standalone editor to create the graphs manually, or rather users can write their own scripts or plug-ins that automate creation of the graphs from their own data. This can be easily achieved by using the simple text-file format FORG3D uses to save the graphs. FORG3D was developed using C++ and uses the OpenGL graphics application programming interface (API). This approach allows FORG3D to take full advantage of the processing capabilities of the modern 3D graphics accelerators, providing high-quality performance for real-time three-dimensional network visualization.

**Figure 1:**
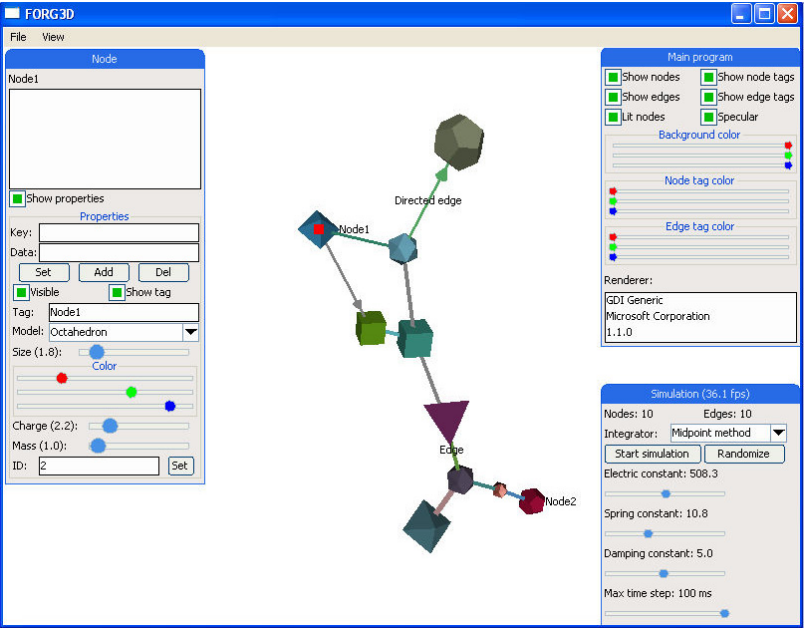
**Graph editor**. Various aspects of the data can be visualized by changing the visual appearance and physical properties of nodes and edges. Graph editor can be used to control these properties, structure of the network and different simulation options.

With the graph editor, users can change the different properties of nodes and edges (Figure 1). The visual properties include visible name, size, color, visibility, node shape and edge direction, while physical properties include mass and charge for the nodes and spring constant for the edges. Both nodes and edges can also be assigned with custom textual properties that can be seen when the object is selected. These can be used to provide users with more information about the object in question, for example if the nodes in the graph would represent proteins and edges would represent protein-protein interactions, the custom node properties could contain protein identifier and description of its function while edge custom properties could contain information about the type of the interaction. Users also have control over different options affecting the overall visual appearance, such as colors and different 3D rendering settings.

Users can also change global simulation options, including electric, spring and damping constants and select a suitable numerical integration method used for the physical simulation. When the simulation is started, the nodes and edges start moving based on their set physical properties and the selected integration method. Edges are assigned with spring constant, and nodes with mass and electric constant. Spring simulation is based on Hooke's law of elasticity where spring constant determines the strength of the connection, and nodes are simulated as electrically charged particles that repel each other determined by their assigned mass and electric constant, based on Coulomb's law.

Based on Coulomb's law the electrostatic force between two charged particles (nodes) can be presented as $F_{c}=\frac{k_{e}q_{1}q_{2}}{r^{2}}$, and the restoring force of a spring based on Hooke's law can be presented as *F*_*h *_= -*k*_*s*_*kr*, where *r *is the distance between two charged particles, *q*_1 _and *q*_2 _are the charges of the particles, *k *is the spring constant of the connecting spring (edge), *k*_*e *_and *k*_*s *_are the global electric and spring constants. When the simulation is running, the particles try to reach a distance where these forces are in equilibrium, this distance can be presented as $r=\sqrt[{3}]{\frac{k_{e}q_{1}q_{2}}{-k_{s}k}}$. The global damping constant representing friction is subtracted from the forces using the following formula *F *= -*kv*, where -*k *is the global damping constant and *v *is the velocity. The simulation works by taking the forces based on Coulomb's and Hooke's law and assigning them to Newton's law of motion *F *= *ma*. Newton's Laws allow one to relate the position, velocity and acceleration of the simulated nodes as a differential equation for the unknown position of the node as a function of time. Numerical integration can therefore be used to solve the differential equation and advance the simulation by a small time step (max time step, adjustable by the user).

The available numerical integration methods in FORG3D are Euler method, Midpoint method and the fourth-order Runge-Kutta method [15]. The purpose of the numerical integration methods is to compute location of the nodes in the simulation, given the affecting forces. The methods differ in their time-complexity and precision, Euler method being the fastest and most inaccurate and therefore suitable for real-time simulation of large graphs with thousands of nodes and edges, while Runge-Kutta is the most complex and accurate method and can be used when higher precision is needed or when large amount of computing power is available. Besides performance, the selection of the numerical integration method also affects the precision and stability of the physical simulation, Runge-Kutta method resulting in most stable graphs where the behavior of the graph is closest to an ideal physical model and will most likely achieve a stable equilibrium of the modeled forces. With small networks, the resulting networks should be close to identical no matter what numerical integration method is used. When the size of the network, and therefore number of the affecting forces, increases, the simpler numerical integration methods may result in networks that do not accurately estimate the affecting forces, producing a network that is not able to reach an equilibrium. This can result in a network where the distances between the nodes do not accurately represent the connection strengths between the nodes, and therefore users should always use the most complex numerical integration method that the size of the network and the amount of computing power allows them to use.

When the simulation is running, these forces are applied to the nodes and edges, pulling the nodes closer or pushing them further apart from each other. The movement of the simulated nodes and edges can be observed in real time while these forces are applied to the simulation iteratively and the movement will continue until equilibrium is reached or the simulation is stopped. Users can interact with the simulation by selecting and dragging nodes around. This also allows for an interactive explorative approach where the connection strength between different nodes can easily be observed by simply dragging a node around and watching how this will affect the connecting nodes. Users can also change their viewpoint by rotating the graphs and zooming in and out, making it easier to inspect specific parts of the graph from different angles and distances.

The graph editor can be used to create the graphs and adjust all the available options, but FORG3D is most useful when automated scripts are used to create the graphs from the experimental data. The text-file format FORG3D uses is simple and easy to use. An example of creating two nodes and a connecting edge is presented here:

NODE:protein1

TAG:P53_HUMAN

SIZE:1

NODE:protein2

TAG:MDM2_HUMAN

SIZE:1

EDGE:protein1, protein2

WIDTH:1

Global simulation parameters and all the other visual and physical properties of nodes and edges can be changed with similar notation.

## Results

To evaluate the performance of FORG3D, simulated network graphs of different sizes were created and tested on various modern desktop and laptop computer setups (Dell 3 GHz, MacBook Pro 2,4 GHz, Toshiba 3 GHz). Performance of physical simulation needed to arrange the network is based on the available CPU processing power and it was observed that a network consisting of thousands of nodes and edges could be simulated in real time. The performance of exploring the network, while the simulation is not running, is based on the OpenGL 3D rendering performance and it was observed that networks with tens of thousands of nodes and edges could be explored while the simulation is stopped. Therefore if the user wants to interact in real time with the network while running the simulation, the size of the network is limited to thousands of nodes and edges, but if there is no need for interaction while running the simulation, the user can have a network of tens of thousands of nodes and edges, run the simulation that arranges the network and then explore it afterwards. How well the network can be interpreted largely depends on the structure of the network and physical properties assigned to nodes and edges, however the ability to rotate and zoom the network in three-dimensions makes it easier for the user to focus on selected parts of the network.

FORG3D implements many favorable features that are generally assigned to force-directed graphs [16,17]. These include good quality of graphs with very good aesthetic properties, such as few edge crossings, uniform node distribution and good symmetry. The behavior of the graph is also intuitive and easy to predict, as it is based on physical properties of common objects such as springs. The graph is also very interactive as the user can observe how the network evolves and arranges itself into a stable configuration, and furthermore, the user can interact with this process by moving parts of the graph or adding or removing nodes/edges. Doing the visualization in three-dimensions further adds to the aesthetic properties and intuitiveness of the graph as it increases the resemblance to actual real-life objects.

### A case study: Visualization of integrated *Caenorhabditis elegans *data

**Figure 2:**
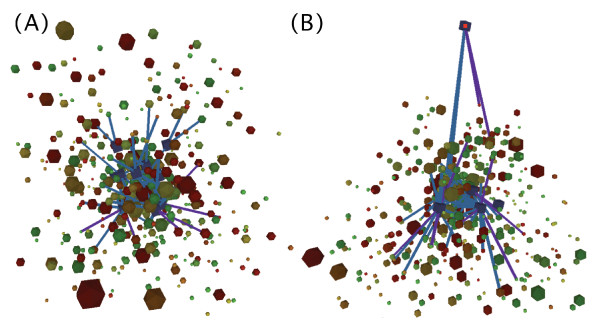
**Integrated *C. elegans *data network**. (A) In the visualized network, nodes represent genes and edges represent interactions between the genes. Measured fold change in gene expression was used to set the color of the nodes, green indicating up regulation, red indicating down regulation and yellow indicating no change between samples. Size of the nodes represents the number of Gene Ontology classes assigned to a gene. Query genes used to create the genetic interaction data are visualized as blue cubes. Width and spring constant of the edges represent interaction strength, and the color represents the screening method that was used to evaluate the interaction between the genes, blue for Byrne et al. and violet for Lehner et al. Because of the large number of the edges, only the edges with strongest interactions were set to visible (interaction strength > 5). (B) The hub node representing the *daf-2 *gene was selected and dragged to the side. Other nodes followed according to the strength of the edges as determined by the spring constant. Notice discrete groups of connected nodes representing genes directly connected to *daf-2*, and genes indirectly connected to *daf-2*. In the actual real-time simulation the movement speed and direction of the nodes are clear indications of the strength and structure of the interaction network.

To demonstrate visualization of actual high throughput genome scale data, a network graph that integrates genomic data from different sources was created. Interaction data was obtained from a study describing genetic interactions *in Caenorhabditis elegans *[9] and combined with a whole genome *C. elegans *gene expression microarray data set obtained from a transgenic Parkinson's Disease model compared to wild type worms [18,19], which was combined with functional gene annotation information from Wormbase [20]. In the resulting network (Figure 2A), nodes represent genes and edges represent interactions between the genes.

The interaction data produced by Byrne et al. [9] was based on generation of synthetic genetic interactions. Query null mutant strains were subjected to RNA interference (RNAi) screens and animals that displayed synthetic phenotypes from RNAi were scored as having interaction between the query gene (11 tested) and the RNAi target (858 tested). In total 1246 interactions were obtained. Similarly, genetic interactions based on RNA interference were defined by Lehner et al. by testing 65,000 gene pairs that found approximately 350 interactions [11]. Interaction strength was defined by degree of genetic interaction, as scored by the observer with a range of 0–6.

When constructing the network, genes that were not available in both interaction and expression data sets were filtered out. This resulted in a network of 449 nodes and 1223 edges. Measured fold change in gene expression between two samples was used to set the color of the nodes, green indicating up regulation, red indicating down regulation and yellow indicating no change between samples. The size of the nodes was set to represent the number of Gene Ontology classes assigned to the genes in question, indicating how well the function of the genes are known and how functionally active they are. Width and spring constant of the edges represent interaction strength, resulting in a network where strongly interacting genes are located closer to each other and connected with wider edges. The color of the edges was set to represent the screening method that was used to evaluate the interaction between the genes, blue for Byrne et al [9]. and violet for Lehner et al. [11]. Because of the large number of the edges, only the edges with strongest interactions (interaction strength > 5) were set to visible. Custom textual properties were added to nodes and edges, representing additional information about the genes and interactions, such as gene annotations from Wormbase. It was observed that network of this size could be simulated in real-time using any of the available physical simulation models, and therefore the most accurate model, Runge-Kutta, was used.

From the visualization, most of the functionally well-known genes, indicated by large node size, are located in the center of the network, which can be explained by the fact that well described genes also are likely to have the strongest known interactions with other genes. This suggests further more detailed study of strongly regulated genes that are either 1) well known and at the outer limits of the network, indicating multiple traceable regulatory pathways or 2) not-well known and in the center of the network, indicating possible novel functional findings. It could also be seen that many genes that were up-regulated (green nodes) were also directly or indirectly networked with down-regulated genes (red nodes). An example is the gene *aph-1 *(up-regulated), which was connected to *daf-4 *(down-regulated) through *glp-1*. This suggests complex changes in gene expression during Parkinson's disease. The real strength of FORG3D in biological interpretation may be in its ability to find key network relationships by taking advantage of its ability to move nodes and explore the dynamic movement of edges that drag other nodes along (Figure 2B). Observing the formation and movement speed of different parts of the network is a useful tool for exploring complex interactions in the data. Using such an approach, it was found that one of the network hubs, *daf-2*, was connected to many of the up- and down-regulated genes directly, and then these genes were further connected to others, thus indicating the importance of the DAF-2 protein in regulating gene expression in this disease model. DAF-2 is a insulin-like receptor that is now known to be a key protein in aging [21].

The example network is distributed with the FORG3D software package.

## Discussion

FORG3D can be used to create intuitive visually pleasant network graphs that users can interact with. Networks can be manually created and altered, and the simple text-file format makes automatic creation of networks an easy task. As FORG3D is open source software, users can also alter it or integrate it with their own software programs. The types of systems biology data that can be visualized using FORG3D includes, but is not limited to, interactions between genes or gene products, signaling transduction, metabolic pathways, functional interactions and evolutionary relationships. In addition, virtually any kind of experimental data that describes correlations or distances between measurements can be visualized using FORG3D.

FORG3D can also complement other bioinformatics tools by allowing the user to build their own integrated data networks and testing hypothesis by interactively exploring effects of movements of one or more nodes [22,23].

Network visualization is a popular topic in the systems biology field, and there are several existing network visualization tools available [13,14,16,17,24-28]. What separates FORG3D from the existing tools is a combination of advanced features, including 1) FORG3D is not limited to any specific type of network data, such as protein-protein interactions [27] or specific species [24], but can be used to visualize any kind of data that can be presented as a network, 2) networks visualized in FORG3D can be fully customized, including changing the visual appearance of individual nodes and edges, as well the physical properties, which allows for detailed visualization of complex network properties, 3) FORG3D contains a network editor that can be used to easily create and edit networks, 4) users can explore and interact with the network in real-time, drag, edit, delete and add nodes/edges and see how this affects the formation of the network, and therefore observe underlying network connections that would not be detectable from a static network that does not allow real-time interaction [25,26,28], 5) FORG3D is open-source, making it possible for users to alter the tool to suit their needs or to integrate it as a part of their own software or analysis pipelines. Advantages of FORG3D also includes that the implementation, which is based on C++ and OpenGL, takes full advantage of the processing capabilities of the modern 3D graphics accelerator hardware and therefore provides significant performance enhancement over many existing tools and plug-ins for network visualization.

When compared to one of the leaders in the field of biological network analysis and visualization, Cytoscape [14], FORG3D offers the following major advantages: 1) Support for 3D visualization of networks. 2D network renderings are flat and with the currently large amounts of systems biology data, the important features of the network can be obscured. While Cytoscape offers several different 2D layout renderings to help visualize the data, the 3D feature of FORG3D lets the user visualize the data from any possible number of x-y-z perspectives without the need to rearrange the network, and without loss of network structure or information. Moreover, the user may choose the perspective to view the network. This allows flexibility in viewing the network and ability to explore the data in 3D without a preconceived notion or hypothesis. This is of particular advantage in dense networks with multiple hubs and large numbers of nodes, where the complexity of the interactions makes it vital to view the data from as many perspectives as possible. 2) FORG3D allows users to observe and interact with the network in real-time, unlike Cytoscape that does not include such functionality. The importance of this feature is the ability to perform "perturbations" to the network and visualize the effects of such actions on the network. Thus, the user may drag a hub in the network, in one direction and see how the other connected nodes or hubs react, or whether new interactions can be observed. Real-time adding, removing or dragging key nodes away from the network and observing the effects on other nodes and network formation based on physical properties such as spring constant values is available in FORG3D. This provides a type of "virtual network perturbation analysis" for the user and is equivalent to testing a hypothesis on the importance of single nodes in the network. Such an application would be critical in evaluating the relevance of knocking out or down genes in pathological processes, and modeling the outcome on other interacting genes and their protein products. To do this in Cytoscape would require such a large number of node, parameter, and rendering iterations, that it would not be feasible under currently available genome scale data sets. 3) Combining the features of 3D visualization, and perturbing the network, and then visualizing results on other nodes in the network in real-time provides a powerful tool to generate and tests hypothesis on the structure of the network. Such a tool is envisaged to help in interpreting systems biology data and their interactions, but may finally provide the insights needed to model correctly complex biology processes. Taken together, FORG3D is not intended to replace Cytoscape as a visualization tool for systems biology, but to complement and extend the tools already available for systems biology researchers. The field is very demanding, and FORG3D provides an additional tool that is intuitive, visual, and easy to manipulate.

There are various other 3D visualization tools available, such as InterViewer [29], GEOMI [30] and BioLayout Express 3D [31]. One of the advantages of FORG3D when compared to InterViewer and other similar biological visualization tools is that FORG3D is not limited to any one kind of data (such as protein-protein interactions with InterViewer), but can be used to visualize any kind of network data. Other advantages over InterViewer, GEOMI and BioLayout Express 3D include the ability to assign custom properties (both visual and physical) to individual nodes and edges, making it easier to visualize and interpret large amounts of information. The main advantage over these tools though is the ability to interact with the network in real-time, as well as the C++ and OpenGL based implementation that takes full advantage of the 3D acceleration hardware, resulting in enhanced performance that cannot be achieved using Java based tools such as Cytoscape, InterViewer, GEOMI or BioLayout Express 3D.

Limitations of FORG3D include that it does not provide support for many of the existing file formats for network data, but this limitation can be overcome by using the flexible text file format used by FORG3D. As FORG3D is open source, users can also add support to file and data formats of their own choosing. FORG3D works best with networks containing up to thousands of nodes and edges, larger networks are likely to be too computing intensive to be explored in real-time and interpretation of them can be difficult.

The FORG3D project website contains additional information about FORG3D, such as details regarding the background and implementation of the software, file format specifications, detailed user manual, and downloads including source code for the software.

## Conclusion

To demonstrate the concept of real-time force directed three-dimensional graphs in visualization of integrated genome scale data and to provide researchers with a practical bioinformatics tool, we have developed open source software called FORG3D, that can be used to visualize complex genome scale data using real-time three-dimensional force directed graphs. FORG3D was then used to visualize a network that integrates different types of genomics data from various sources. We believe that FORG3D is a useful tool for visualizing and exploring integrated genome scale data.

## Availability and requirements

**Project name**: FORG3D

**Project home page**: 

**Operating system(s)**: Windows. Portable to other operating systems.

**Programming language**: C++

**Other requirements**: OpenGL support.

**License**: Open source. Free for academic and non-academic use.

**Any restrictions to use by non-academics**: None.

## Authors' contributions

JP conceived and carried out the project. GW supervised the project and helped to draft the manuscript. All authors read and approved the final manuscript.
